# FLASH proton density imaging for improved surface coil intensity correction in quantitative and semi-quantitative SSFP perfusion cardiovascular magnetic resonance

**DOI:** 10.1186/s12968-015-0120-6

**Published:** 2015-02-17

**Authors:** Sonia Nielles-Vallespin, Peter Kellman, Li-Yueh Hsu, Andrew E Arai

**Affiliations:** National Heart Lung and Blood Institute (NHLBI), National Institutes of Health (NIH), DHHS Bethesda, MD, USA

**Keywords:** Myocardial perfusion, Quantitative perfusion, Cardiovascular magnetic resonance, Surface coil correction, Proton density frames, Balanced Steady State Free Precession (SSFP), Fast Low Angle SHot (FLASH)

## Abstract

**Background:**

A low excitation flip angle (α < 10°) steady-state free precession (SSFP) proton-density (PD) reference scan is often used to estimate the B_1_-field inhomogeneity for surface coil intensity correction (SCIC) of the saturation-recovery (SR) prepared high flip angle (α = 40-50°) SSFP myocardial perfusion images. The different SSFP off-resonance response for these two flip angles might lead to suboptimal SCIC when there is a spatial variation in the background B_0_-field. The low flip angle SSFP-PD frames are more prone to parallel imaging banding artifacts in the presence of off-resonance. The use of FLASH-PD frames would eliminate both the banding artifacts and the uneven frequency response in the presence of off-resonance in the surface coil inhomogeneity estimate and improve homogeneity of semi-quantitative and quantitative perfusion measurements.

**Methods:**

B_0_-field maps, SSFP and FLASH-PD frames were acquired in 10 healthy volunteers to analyze the SSFP off-resonance response. Furthermore, perfusion scans preceded by both FLASH and SSFP-PD frames from 10 patients with no myocardial infarction were analyzed semi-quantitatively and quantitatively (rest n = 10 and stress n = 1). Intra-subject myocardial blood flow (MBF) coefficient of variation (CoV) over the whole left ventricle (LV), as well as intra-subject peak contrast (CE) and upslope (SLP) standard deviation (SD) over 6 LV sectors were investigated.

**Results:**

In the 6 out of 10 cases where artifacts were apparent in the LV ROI of the SSFP-PD images, all three variability metrics were statistically significantly lower when using the FLASH-PD frames as input for the SCIC (CoV_MBF-FLASH_ = 0.3 ± 0.1, CoV_MBF-SSFP_ = 0.4 ± 0.1, p = 0.03; SD_CE-FLASH_ = 10 ± 2, SD_CE-SSFP_ = 32 ± 7, p = 0.01; SD_SLP-FLASH_ = 0.02 ± 0.01, SD_SLP-SSFP_ = 0.06 ± 0.02, p = 0.03). Example rest and stress data sets from the patient pool demonstrate that the low flip angle SSFP protocol can exhibit severe ghosting artifacts originating from off-resonance banding artifacts at the edges of the field of view that parallel imaging is not able to unfold. These artifacts lead to errors in the quantitative perfusion maps and the semi-quantitative perfusion indexes, such as false positives. It is shown that this can be avoided by using FLASH-PD frames as input for the SCIC.

**Conclusions:**

FLASH-PD images are recommended as input for SCIC of SSFP perfusion images instead of low flip angle SSFP-PD images.

## Background

Signal intensity variations due to surface coil B1-field inhomogeneity affect both quantitative and qualitative analysis of perfusion cardiovascular magnetic resonance (CMR) [1-4]. Several surface coil intensity correction (SCIC) approaches have been implemented [2,3,5-8]. In order to ensure image registration, proton density (PD) weighted images can be acquired as part of the myocardial perfusion imaging sequence, at the initial frames just prior to the contrast agent injection [1]. These PD images are acquired without saturation recovery (SR) preparation and using a low readout flip angle (α < 10°).

Saturation recovery in combination with a steady state free precession (SSFP) readout have been shown to have higher signal and contrast to noise ratio than both gradient-echo echo-planar imaging (GRE-EPI) and fast low angle shot (FLASH) protocols for myocardial perfusion imaging [1] and have produced good results in several clinical studies [9-11]. While the SR-prepared T1-weighted SSFP myocardial perfusion imaging protocol uses a high flip angle (α ~ 50°), the proton density weighted SSFP protocol uses a low flip angle (α < 10°).

The hypotheses of this study were the following. First, the different SSFP off-resonance response of the SSFP PD images due to the low flip angle used might lead to suboptimal surface coil correction when there is a spatial variation in the background B_0_-field. As a consequence, SCIC with low flip angle SSFP images as input might lead to inaccurate semi-quantitative and fully quantitative perfusion measurements. On the other hand, the off-resonance frequency response of FLASH is flat. Therefore, replacing the low flip angle SSFP PD frames by FLASH PD frames in the SSFP perfusion protocol should make the SCIC insensitive to spatial variations in the background B_0_-field and should lead to more accurate semi-quantitative and fully quantitative perfusion measurements.

To address these hypotheses, a new sequence was implemented which acquired low flip angle FLASH PD images followed by SR-prepared T1-weighted high flip angle SSFP perfusion images. B_0_ field maps, SSFP and FLASH PD frames were acquired in 10 healthy volunteers and simulations were performed to analyze the SSFP off-resonance response. Furthermore, perfusion scans preceded by both FLASH and SSFP PD frames from 10 patients with no myocardial infarction were analyzed semi-quantitatively and quantitatively (rest n = 10 and stress n = 1). Intra-subject myocardial blood flow (MBF) coefficient of variation (CoV) over the whole left ventricle (LV), as well as intra-subject peak contrast (CE) and upslope (SLP) standard deviation (SD) over 6 LV sectors were investigated.

## Methods

The existing clinical SSFP perfusion sequence acquired low flip angle (α = 8**°**) SSFP PD images followed by saturation prepared high flip angle (α = 50**°**) SSFP perfusion images. A new sequence was implemented which acquired low flip angle (α = 5**°**) FLASH PD images followed by saturation prepared high flip angle SSFP perfusion images.

### CMR Data acquisition

Imaging was performed on a 3T Siemens MAGNETOM Skyra (Siemens Healthcare, Erlangen, Germany) using a phased array Siemens torso coil and spine array combination typically adding up to 30 channels. In every scanning session a second order shim was performed over a local volume (box) encompassing the whole heart.

Field maps were acquired using a breath-hold multi-echo Dixon fat-water separation technique [12] with the following protocol parameters: readout bandwidth (BW) = 977 Hz/pixel, TE = 1.56, 2.72, 3.88 and 5.04 ms, TR = 14.3 ms, flip angle = 12°, field of view (FOV) = 380 × 285 mm, image matrix = 256 × 192, views per segment = 20, slice thickness = 8 mm, and breath-hold duration 16 heartbeats.

The following parameters were used for the SSFP myocardial perfusion protocol: composite saturation recovery pulse design = 90° [1], readout flip angle α = 50°, TR = 2.5 ms, TE = 1.02 ms, TI = 120 ms, fat suppression, BW = 1085 Hz/pixel, slice thickness = 8 mm, acquisition matrix = 192 × 111, FOV = 380 × 285 mm, parallel imaging with rate 3 TGRAPPA [13]. At the start of each of these non-contrast perfusion acquisitions, 3 SSFP PD weighted images (α = 8°) and 3 FLASH PD weighted images (α = 5°) were acquired using the same imaging parameters described above, except no saturation preparation pulse.

### Human Subjects

All studies were performed under procedures and protocols approved by the Institutional Review Board of the National Institutes of Health (ClinicalTrials.gov identifier NCT00027170).

Ten healthy volunteers were recruited to study the bias introduced by the uneven frequency response of the low flip angle SSFP PD protocol. No contrast agent was used for these experiments.

Ten patients with no myocardial infarction were studied at rest (n = 10) and stress (n = 1) to assess homogeneity of semi-quantitative and fully quantitative perfusion measurements. Gadobutrol (Gadovist, BAY86-4875) was injected during the stress and rest first pass perfusion imaging (0.05 mmol/kg body weight (BW) as bolus injection at stress followed by 0.05 mmol/kg BW as bolus injection at rest, with a total dose of 0.1 mmol/kg BW). Stress perfusion CMR was performed approximately 3 minutes after starting an infusion of adenosine (140 micrograms/kg/min) and rest imaging was done 20 minutes later.

### SSFP off-resonance frequency response

#### Simulation of SSFP off-resonance frequency response

To demonstrate the different SSFP off-resonance response for the α = 8° SSFP PD images and the α = 50° SSFP perfusion images, a Bloch equations simulation was implemented using the protocol parameters described above. The simulated SSFP responses used values of native myocardial T_1_ = 1100 ms and T_2_ = 45 ms, and were not sensitive to the precise value of T_1_ in a range expected for myocardial T1 pre-contrast, which is when PD images are obtained. Image analysis and simulations were performed using Matlab (Mathworks, Inc., Natick, MA, USA).

#### Experimental confirmation of SSFP off-resonance frequency response

In order to translate from theory to experimental confirmation, the signal intensity on the low flip angle SSFP PD images was measured as a function of frequency in 10 healthy volunteers. The center frequency was varied between −200 Hz and +200 Hz in steps of 50 Hz. At each of these 9 frequencies, field maps, 3 SSFP PD and 3 FLASH PD frames, as well as 3 non-contrast perfusion SSFP frames, were acquired for one mid-ventricular short axis slice with the protocol parameters described above.

The measured SSFP PD frequency response was derived as follows. Averaging the 3 SSFP PD images and the 3 FLASH PD images respectively created a mean SSFP PD image and a mean FLASH PD image for each center frequency. Signal intensity was measured using ROIs placed in the septum of the mean SSFP and mean FLASH PD image at each center frequency in the 10 healthy volunteers. Frequency was measured off B_0_ field maps in the same part of the heart. The signal from the SSFP PD images was normalized with the signal from the FLASH PD images. The resulting signal was plotted against the frequency measured from the B_0_ field maps. These experimental results were plotted against the Bloch simulation of the SSFP PD protocol frequency response.

### Effect of FLASH and SSFP PD images on semi-quantitative and quantitative perfusion measurements

Rest SSFP perfusion scans preceded by 3 SSFP PD and 3 FLASH PD frames were performed in 10 patients with no myocardial infarction. Three slices were acquired with the protocol parameters described above. To compare the SCIC performance using either FLASH PD or SSFP PD images as input, the most basal slice of each of the 10 rest perfusion scans was motion corrected [14], after which both a semi-quantitative and a fully quantitative perfusion analysis were performed. Image analysis was performed using IDL (Interactive Data Language, Exelis Visual Information Solutions, Boulder, CO, USA).

Scans were divided into two groups: scans where artifacts were visually apparent in the LV region-of-interest (ROI) of the SSFP PD images, and scans where no apparent artifacts were present in the LV ROI of the SSFP images. Average inter-subject measures and intra-subject variability were calculated for each group and each PD technique.

For the semi-quantitative analysis, the endocardial and epicardial borders of the perfusion images were manually traced and registered. The myocardial regions of interest were divided into six circumferential sectors at each slice location. Time signal intensity curves of the blood cavity and the myocardial sectors were generated, after using either the FLASH PD or SSFP PD reference signal intensity for surface coil intensity normalization. Time signal intensity upslope (SLP) and peak myocardial contrast enhancement (CE) were computed as described in previous works [6,7]. Results were expressed as mean ± standard deviation (SD). Inter-subject mean CE and SLP were calculated. A two-tailed paired *t*-test of the intra-subject SD of each of these regional perfusion measures was used to compare their variability across the six regions of the LV.

For the fully quantitative analysis, the perfusion images were surface coil intensity corrected using either the FLASH PD or SSFP PD images as input. After that, myocardial blood flow (MBF) was quantified on a pixel-by-pixel basis using a Fermi-constrained deconvolution algorithm [15]. Results were expressed as mean ± SD. The inter-subject mean MBF was calculated. The CoV of the MBF estimates in the LV was evaluated in these quantitative perfusion maps and a two-tailed paired *t*-test was used to compare the homogeneity of the maps in the LV region of interest.

A stress perfusion scan was also semi-quantitatively and fully quantitatively analyzed but only summarized as a figure due to the sample size (n = 1).

## Results

### SSFP off-resonance frequency response

**Figure 1:**
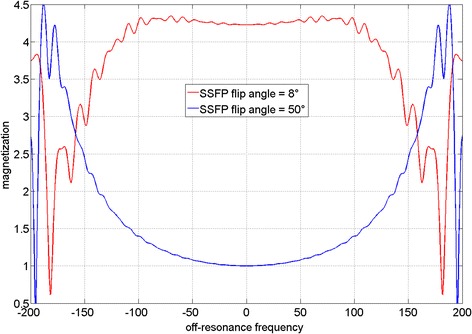
**Simulated off-resonance frequency response for SSFP protocols with a flip angle α = 8°, representing the low flip angle SSFP PD weighted acquisition, and a flip angle of α = 50°, as used in the high flip angle SSFP perfusion acquisition.** The frequency response of the α = 50° protocol is quite flat over a range of ± 100 Hz, while the frequency response of the α = 8° protocol shows a deviation of 40% at ± 100 Hz. It can be observed that the banding artifacts, which occur due to off-resonance, exhibit much brighter edges at low flip angles than at high flip angles.

The Bloch equations simulations of the off-resonance frequency response for SSFP protocols with a flip angle α = 8° and a flip angle α = 50° are shown in Figure 1. The frequency response of the high flip angle (α = 50°) SSFP perfusion protocol is flat over a range of ± 100 Hz, while the frequency response of the low flip angle (α = 8°) PD SSFP protocol shows a deviation of about 40% at ± 100 Hz relative to on resonance. Furthermore, it can be observed that the banding artifacts, which occur due to off-resonance, exhibit much brighter edges at low flip angles than at high flip angles.

**Figure 2:**
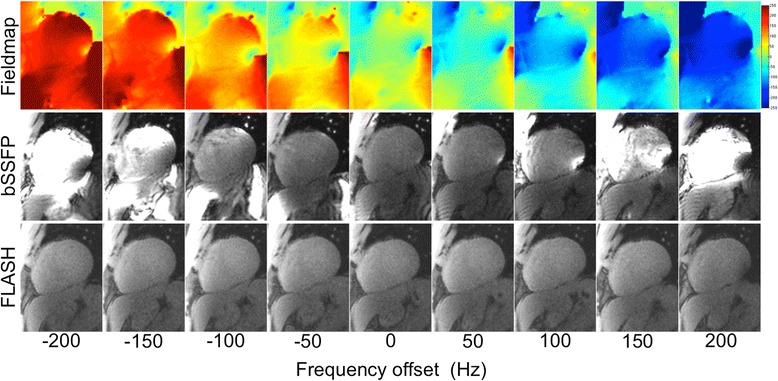
**B**_**0**_ **field maps, low flip angle SSFP PD and FLASH PD short axis images for frequency offsets ranging from −200 Hz to 200 Hz in a healthy volunteer.** The sensitivity to off-resonance of the SSFP protocol can be observed in the form of inhomogeneous signal intensity and banding artifacts, while the FLASH images are artifact free.

**Figure 3:**
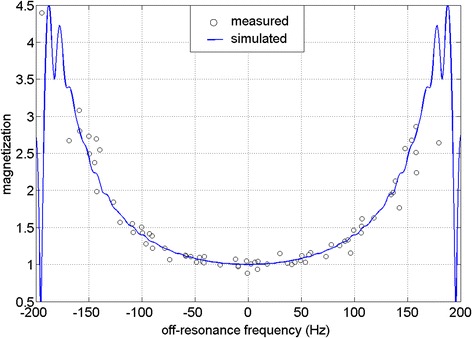
**Bloch equations simulation (blue line) and measured (black circles) SSFP off-resonance frequency response for a flip angle α = 8° in 10 volunteers.** The measured and simulated data agree reasonably well.

Figure 2 shows an example volunteer data set, consisting of field maps, SSFP PD images and FLASH PD images for frequency offsets ranging from −200 Hz to 200 Hz. The high sensitivity of the SSFP protocol to off-resonance leads to banding artifacts on the myocardium in all but the center frequency frame, while the FLASH images are artifact free across all frequencies. The SSFP PD images frequency response derived from 10 volunteers agrees reasonably well with the simulated SSFP frequency response (Figure 3).

### Effect of FLASH and SSFP PD images on semi-quantitative and quantitative perfusion measurements

**Figure 4:**
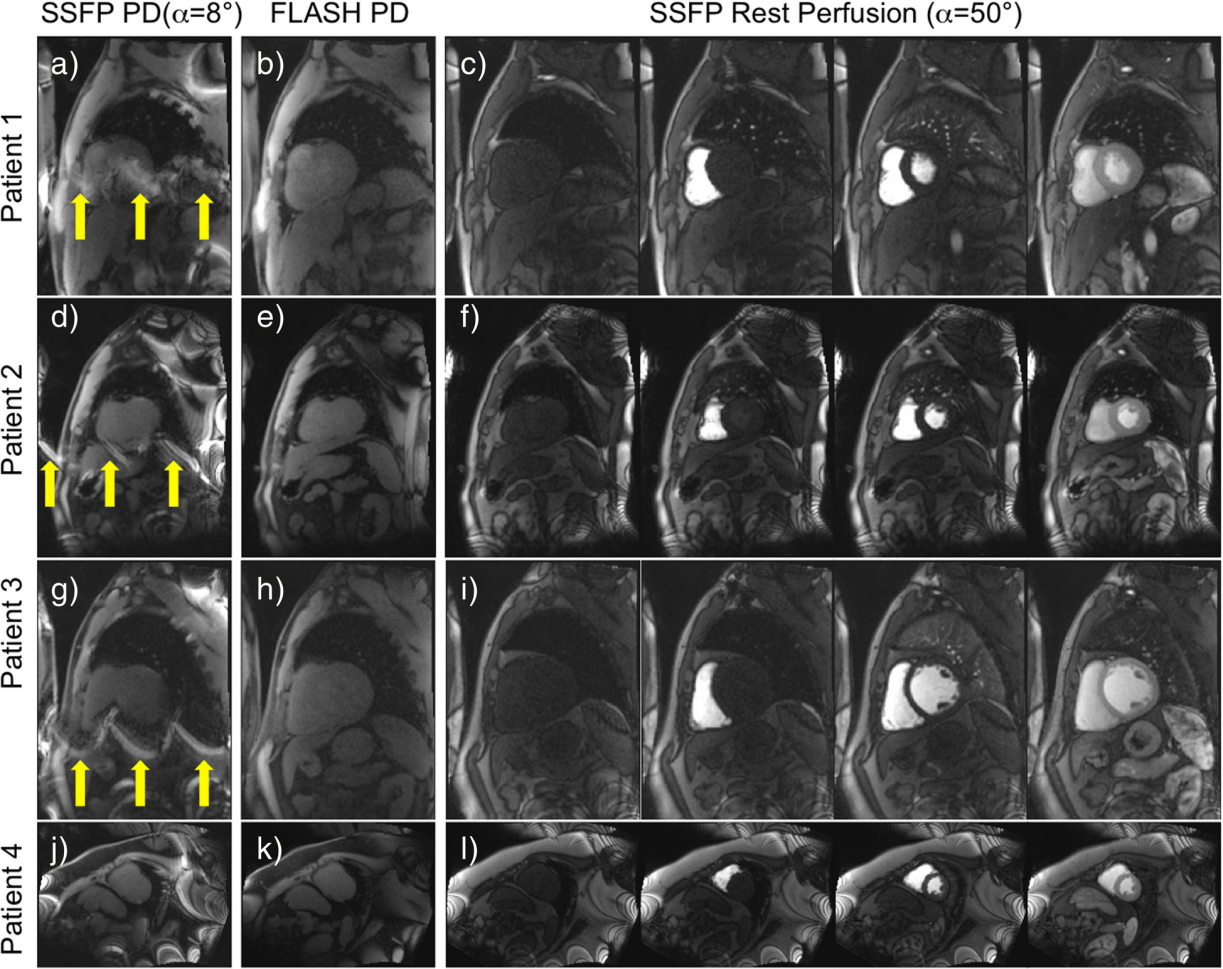
**Full field of view rest scans in 4 patients with no myocardial infarction.** In the first two patients, the low flip angle SSFP PD frames contain banding artifacts on top of the myocardial region of interest (yellow arrows in images **a**, **d** and **g**), while the high flip angle SSFP perfusion frames **(c, f and** **i)** and the FLASH PD frames **(b, e and** **h)** do not. These ghosting artifacts repeat themselves 3 times, which is consistent with the parallel imaging acceleration factor of 3 used for these acquisitions. In the fourth patient, none of the images contain banding artifacts on top of the LV region of interest **(j, k and l)**.

Selected frames from example rest scans in four patients using the SSFP perfusion protocol preceded by both FLASH and SSFP PD frames are shown in Figure 4. In three cases, the banding artifacts on top of the myocardial region of interest are severe in the low flip angle SSFP PD images. These ghosting artifacts repeat themselves 3 times, which is consistent with the parallel imaging acceleration factor of 3 used for these acquisitions. Neither the high flip angle SSFP perfusion images nor the FLASH PD images exhibit these ghosting artifacts despite the same parallel imaging factor of 3.

**Figure 5:**
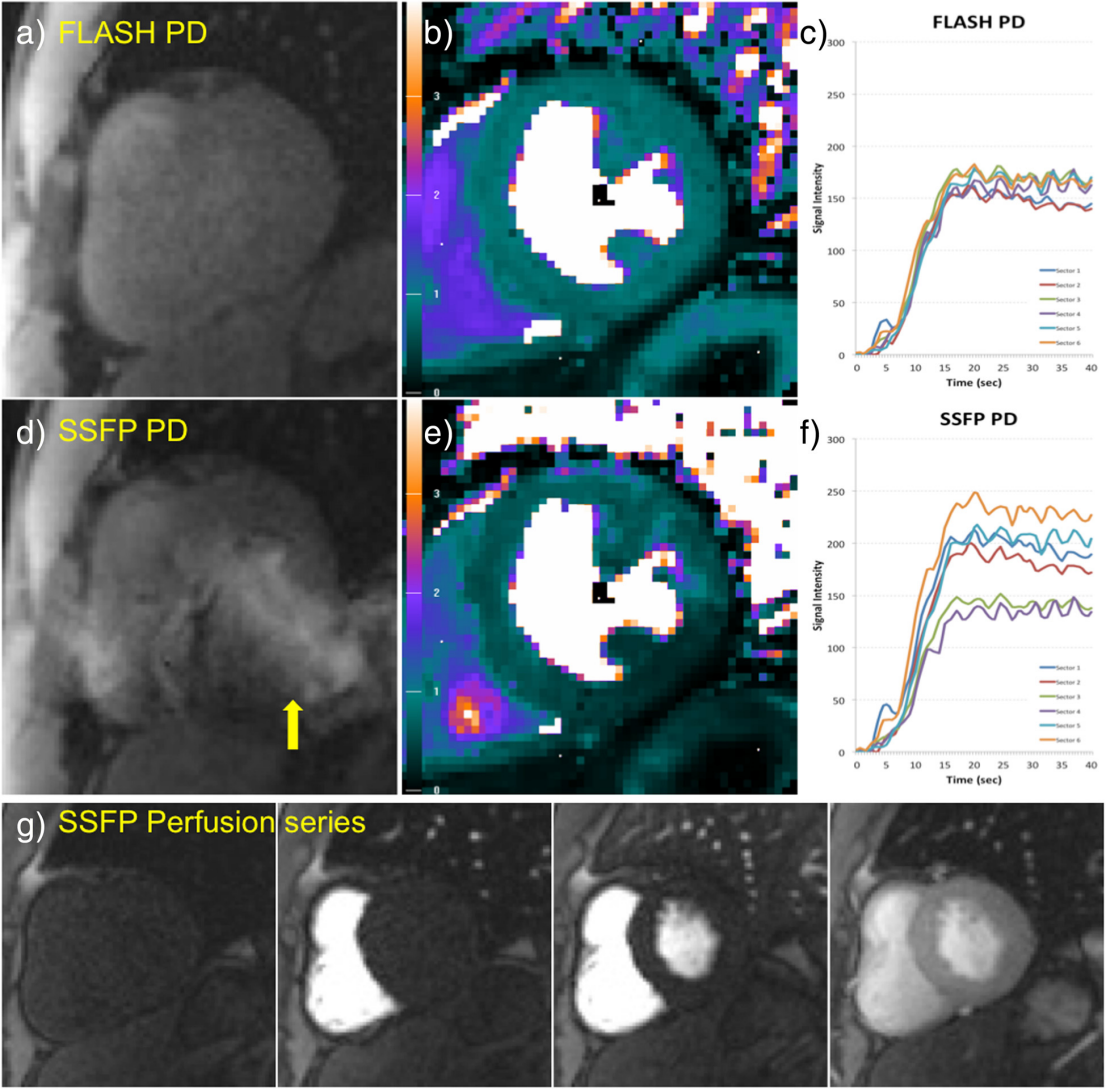
**Example rest scan in a patient with no myocardial infarction.** FLASH **(a)** and low flip angle SSFP **(d)** PD frames and their corresponding quantitative MBF maps (**b** and **e** respectively) and regional perfusion signal intensity curves from six sectors of the LV **(c and f)**, as well as some representative frames from the high flip angle perfusion series **(g)**. The low flip angle SSFP PD image **(d)** shows banding artifacts on top of the myocardium (yellow arrows), which lead to overcorrection of the SCIC, causing artefactual lower perfusion values in the inferior and inferolateral walls **(e)**. This affects both the MBF maps and in the regional time intensity curves **(f)**. The FLASH PD frames **(a)** are unaffected by banding artifacts, which leads to an improved SCIC, a more homogeneous MBF map **(b)** and better agreement between the regional time intensity curves **(c)**.

**Figure 6:**
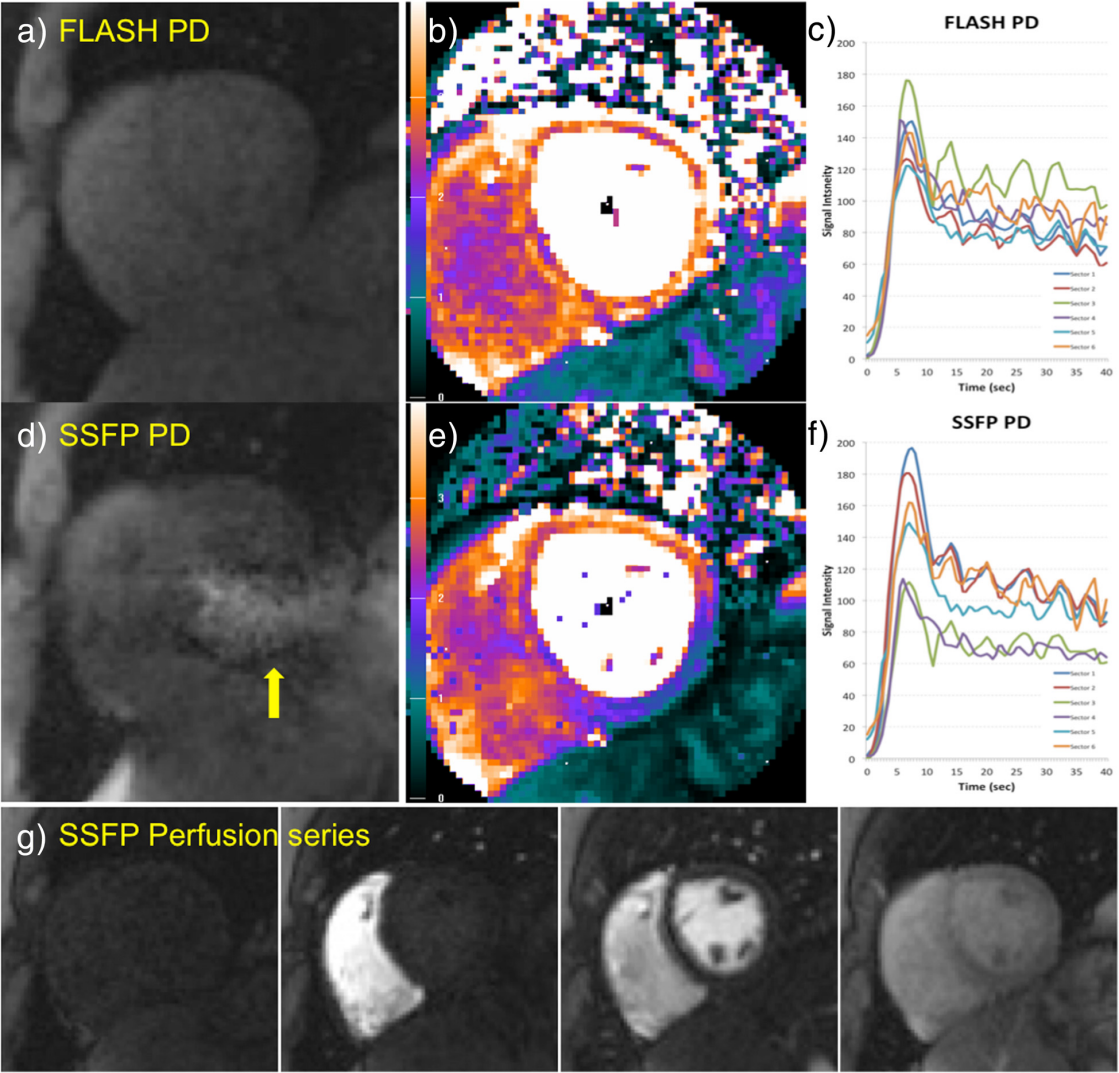
**Example stress scan in a patient with no myocardial infarction and no coronary artery stenosis.** FLASH **(a)** and low flip angle SSFP **(d)** PD frames and their corresponding quantitative MBF maps (**b** and **e** respectively) and regional perfusion signal intensity curves **(c and f)**, as well as some representative frames from the high flip angle perfusion series **(g)**. The low flip angle SSFP PD image **(d)** shows banding artifacts on top of the myocardium (yellow arrows), which lead to overcorrection of the SCIC, causing artefactual lower perfusion values in the inferior and inferolateral walls **(e)**. This affects both the MBF map, and the regional time intensity curves **(f)**, and could lead to a false positive diagnosis. The FLASH PD frames **(a)** are unaffected by banding artifacts, which leads to an improved SCIC, a more homogeneous perfusion map **(b)** and better agreement between the perfusion curves **(c)**.

Figures 5 and 6 show one example rest scan (Figure 5) and one example stress scan (Figure 6) in two patients with no myocardial infarction. While the high flip angle SSFP perfusion frames are artifact free in the myocardial region of interest (Figures 5g, 6g), the low flip angle SSFP PD images (Figures 5d, 6d) exhibit severe artifacts across the LV ROI. These artifacts lead to overcorrection in the SCIC, causing artefactual lower MBF values in the inferior and inferolateral segments (Figures 5e, 6e), and residual heterogeneity of regional perfusion time intensity curves (Figures 5f, 6f). On the other hand, the FLASH PD frames (Figures 5a, 6a) are unaffected by banding artifacts, which leads to an improved SCIC, a more homogeneous perfusion map (Figures 5b, 6b), and less sector to sector heterogeneity of the regional perfusion time intensity curves (Figures 5c, 6c).

The results of both the semi-quantitative and fully quantitative perfusion measures over 10 rest perfusion scans in patients with no myocardial infarction, surface coil intensity corrected either with FLASH or SSFP PD images, are shown in Table 1. Artifacts were apparent in the LV ROI of the SSFP PD images in 6 out of the 10 scans. In subjects with artifacts on the SSFP PD images, all three variability metrics were statistically significantly lower (better) when using the FLASH PD frames as input for the surface coil inhomogeneity estimate (Table 1). In the cases where artifacts were not visually apparent in the LV ROI of the SSFP PD images, there were no statistically significant differences and similar mean measures of variability were found for all metrics (Table 1).

**Table 1:** **Comparison of quantitative and semi-quantitative perfusion measures over 10 rest perfusion scans in patients with no myocardial infarction, surface coil corrected either with FLASH or SSFP PD frames**

		**Inter-subject MEAN**		**Intra-subject CoV or SD**		**p**
		**FLASH PD**	**SSFP PD**	**FLASH PD**	**SSFP PD**	
APPARENT ARTEFACTS in LV ROI in SSFP PD images (6/10)	MBF (ml/min/g)	0.98 ± 0.18	0.87 ± 0.13	0.3 ± 0.1	0.4 ± 0.1	0.03
	Peak enhancement (A.U.)	134 ± 67	140 ± 65	10 ± 2	32 ± 7	0.01
	Upslope (A.U.)	0.25 ± 0.09	0.25 ± 0.11	0.02 ± 0.01	0.06 ± 0.02	0.03
NO APPARENT ARTEFACTS in LV ROI in SSFP PD images (4/10)	MBF (ml/min/g)	1.06 ± 0.25	1.05 ± 0.27	0.25 ± 0.13	0.25 ± 0.14	0.5
	Peak enhancement (A.U.)	139 ± 75	140 ± 70	8 ± 3	7 ± 2	0.25
	Upslope (A.U.)	0.35 ± 0.12	0.35 ± 0.11	0.04 ± 0.02	0.04 ± 0.02	0.55

Results are presented for two groups: scans where artifacts were apparent in the left ventricular (LV) region-of-interest (ROI) of the SSFP PD images (6 out of 10), and scans where no apparent artifacts were present in the LV ROI of the SSFP images. Inter-subject mean myocardial blood flow (MBF), peak myocardial contrast enhancement (CE) and time intensity upslope (SLP) are presented. For method comparison, intra-subject MBF coefficient of variation (CoV) over the entire LV, as well as intra-subject CE and SLP standard deviation (SD) over 6 LV sectors, are presented together with the results of the paired *t*-test comparison. In the data pool where artifacts were apparent in the SSFP PD images, all variability parameters for all three measures were statistically significantly smaller for the data surface coil intensity corrected with FLASH PD images. In the data pool where artifacts were not apparent in the SSFP PD images, all measures were in close agreement.

## Discussion

The first hypothesis of this study was that low flip angle SSFP PD images might lead to suboptimal SCIC when there is a spatial variation in the background B_0_-field. Two sources of error were identified. First, the low flip angle SSFP PD images are modulated both by surface coil inhomogeneity and by their own off-resonance frequency response. Second, the low flip angle SSFP protocol can exhibit severe ghosting artifacts originating from off-resonance banding artifacts at the edges of the field of view that parallel imaging is not able to unfold, which are not present in the high flip angle SSFP perfusion acquisition.

Figure 1 shows the different frequency responses of a high flip angle (α = 50°) and a low flip angle (α = 8°) SSFP protocol. While the frequency response of high flip angle SSFP protocol is quite flat over a range of ±100 Hz, the frequency response of the low flip angle SSFP protocol shows a deviation of 40% at ±100 Hz. Figures 2 and 3 show how the low flip angle SSFP PD images are modulated both by surface coil inhomogeneity and by their own off-resonance frequency response. The off-resonance variation in the left ventricle (LV) at 1.5 T and 3 T after shimming over a local heart volume has been previously measured [16]. At 3 T, the mean off-resonance frequency in the LV myocardium was 15.4 ± 29.3 Hz. The maximum off-resonance in the LV was 125.0 ± 40.6 Hz (n = 18). It can be inferred from the plot in Figure 3 that in patients where the off-resonance is up to 100 Hz, the normalization by the bSSFP PD could potentially bias the data by up to a ratio of 1.4 to 1, providing suboptimal SCIC.

In addition, SSFP protocols suffer from banding artifacts, especially at 3 T where off-resonance effects due to susceptibility at tissue interfaces are more severe than at 1.5 T [17]. These SSFP off-resonance banding artifacts exhibit bright edges at low flip angles (Figure 1). Therefore, for wide bore systems with a high gradient in off-resonance variation at the edges of the field of view (FOV), low flip angle SSFP PD images will exhibit be much brighter signal bands at the edge of the FOV. The k-space for these local regions will have a chemical shift in the readout direction. Thus, k-space methods that calculate a global set of coefficients will have an inconsistency between regions with and without chemical shift [18]. This may result in parallel imaging artifacts that manifest as residual ghosting in the phase encode direction due to errors in the coefficients. These artifacts are demonstrated in Figures 4, 5 and 6 (yellow arrows). It can be observed that these banding artifacts are repeated three times, as would be expected from a protocol with parallel imaging acceleration factor of 3. It can also be observed that these artifacts are not present in the high flip angle SSFP perfusion images. Although image domain methods should be able to unwrap this data correctly, these methods are currently less robust to FOV wrap and respiratory motion, both of which are usually present in free breathing myocardial perfusion scans. Figures 5 and 6 demonstrate that these artifacts can lead to errors in the SCIC, which then lead to errors in the quantitative perfusion maps and the regional semi-quantitative perfusion indexes. In particular, Figure 6 shows a case where these artifacts lead to a false positive in a stress perfusion scan. It is also shown that this can be avoided by using FLASH PD frames as input for the SCIC.

While the ideal solution to the former problem would be to achieve a shim that would effectively eliminate off-resonance over the myocardial region of interest, the latter problem of the ghosting artifacts caused by banding at the edges of the FOV which the parallel imaging algorithm fails to unfold would require a shim that would effectively eliminate off-resonance over the entire FOV. This is not currently feasible in a clinical setting with state of the art clinical scanners, and therefore alternative solutions must be found. Alternatively, manually changing the center frequency might move these bands away from the myocardial region of interest for the low flip angle SSFP PD frames. However, the need to run several test scans to ensure artifact free images would increase complexity, extend the examination time and worsen the clinical workflow. Since the off-resonance frequency response of FLASH is flat, low flip angle FLASH PD images are primarily modulated by the surface coil inhomogeneity and do not suffer from banding artifacts as in SSFP. Therefore, by replacing the low flip angle SSFP PD frames in the SSFP perfusion protocol by FLASH PD frames, both the banding artifacts due to off-resonance and the artefactual signal modulation due to off-resonance frequency response in the surface coil intensity correction can be avoided.

Even though the sample size in this study (n = 10) was small, statistically significant differences in MBF, CE and SLP could be observed when using either FLASH or low flip angle SSFP PD frames for SCIC. The results show that, when artifacts are not visually apparent in the low flip angle SSFP PD images, both techniques produce similar results. On the other hand, when artifacts are visually apparent in the low flip angle SSFP PD images, FLASH PD images produce more homogeneous quantitative MBF maps and reduced variation in semi-quantitative perfusion indexes (CE and SLP).

## Conclusions

Low flip angle SSFP PD images are susceptible to artifacts due to B_0_ inhomogeneities in the myocardial regions of interest. Also, low flip angle SSFP PD images can exhibit severe ghosting artifacts originating from off-resonance banding artifacts at the edges of the field of view that parallel imaging is not able to unfold, which are not present in the high flip angle SSFP perfusion acquisition. Using FLASH PD images avoids both SSFP-related artefactual mechanisms in the presence of off-resonance and improves perfusion quantification. Thus, FLASH PD images are recommended for surface coil intensity correction of SSFP perfusion images in place of low flip angle SSFP PD images.

## Abbreviations

α: Flip angle
CMR: Cardiovascular magnetic resonance
FLASH: Fast Low Angle SHot
GRE-EPI: Gradient-Echo Echo Planar Imaging
LGE: Late gadolinium enhancement
LV: Left ventricle
PD: Proton density
ROI: Region of iInterest
SAR: Specific absorption rate
SCIC: Surface coil intensity correction
SR: Saturation recovery
SSFP: Balanced steady state free precession
